# Engineering and Precision Delivery Strategies of Functionalized Extracellular Vesicles for Antitumor Therapy

**DOI:** 10.3390/pharmaceutics18101215

**Published:** 2026-09-24

**Authors:** Xuan Zhang, Xiaowei Zhou, Shiqing Fu, Xinhua Liu, Yanfei Xie, Yue Zhang, Yanyun Fang, Jun Yu

**Affiliations:** 1 Translational Medicine Center, Jiangxi University of Chinese Medicine, Nanchang 330004, China20070945@jxutcm.edu.cn (Y.X.); 2 Jiangsu Key Laboratory for Pharmacology and Safety Research of Chinese Materia Media, School of Pharmacy, Nanjing University of Chinese Medicine, Nanjing 210023, China; 3 Lemole Center for Integrated Lymphatics and Vascular Research, Department of Cardiovascular Sciences, Lewis Katz School of Medicine, Temple University, Philadelphia, PA 19140, USA; jun.yu@temple.edu

**Keywords:** extracellular vesicles, functional modification, targeted recognition, precision delivery, antitumor therapy

## Abstract

Nanomedicine harnesses the unique benefits of nanocarriers to markedly enhance drugs targeting tumors, which is becoming a promising approach to achieve precise and effective antitumor therapy. Extracellular vesicles (EVs), natural nanoscale entities, have garnered extensive attention due to their high biocompatibility and low immunogenicity. However, they often lack the specificity to identify and target lesion sites, leading to side effects when used systemically. Thus, modifying EVs to enhance their targeting ability is crucial. This review comprehensively elucidates the use of natural EVs in tumor treatment and functionalization strategies for EVs, including bioengineering modifications of parent cells, covalent and noncovalent modifications for the EV surface. The particular emphasis is placed on the precision delivery strategies of these functionalized EVs. According to the different delivery endpoints, they can be classified into tumor microenvironment delivery, intracellular delivery into tumor cells, and dual delivery to the tumor microenvironment and the tumor cell. Future perspectives focus on the essential aspects of constructing functionalized EVs and anticipate their evolving role in tumor-targeted therapies. Overall, this review offers a thorough analysis of recent advancements in functionalized EVs for antitumor therapy, providing valuable insights for their future development and precision biomedical applications.

## 1. Introduction

Conventional cytotoxic chemotherapy has long served as the cornerstone of comprehensive tumor treatment; nevertheless, insufficient effective drug accumulation within tumor lesions and severe systemic toxicities remain primary bottlenecks restricting clinical therapeutic efficacy [1]. Meanwhile, the nonselective biodistribution of chemotherapeutic agents across healthy organs triggers adverse effects, including myelosuppression, gastrointestinal irritation, and cardiac, hepatic and renal injury, which further limits the allowable therapeutic dosage and treatment cycle [2]. Accordingly, engineering precision tumor-targeted delivery platforms that optimize systemic drug distribution, boost intratumoral drug exposure in malignant cells, and minimize off-target injury to normal tissues constitutes a critical strategy to elevate the therapeutic index of cancer treatment.

Precision delivery platforms provide new opportunities to improve therapeutic efficacy by tuning drug biodistribution, tumor tissue accumulation, cellular uptake, and intracellular cargo release behavior. Cancer nanomedicine leverages synthetic nanocarriers to encapsulate and deliver therapeutic agents, which reduces nonspecific circulation loss of free drugs, improves drug chemical stability, and enhances local drug utilization within solid tumor lesions [3]. However, the in vivo delivery efficiency of synthetic nanocarriers in solid tumors remains far from satisfactory. Previous systematic statistical analysis has demonstrated that only approximately 0.7% of the total injected nanoparticle dose ultimately accumulates in solid tumors. Abnormal tumor vasculature, dense extracellular matrix, intrinsic tumor heterogeneity, and rapid clearance mediated by the mononuclear phagocyte system jointly hinder efficient tumor-targeted delivery [4]. Therefore, an ideal tumor delivery platform cannot merely rely on passive tumor accumulation; instead, it requires comprehensive optimization covering multiple sequential delivery processes: long-term circulatory stability, deep tumor tissue penetration, specific cellular recognition, orderly intracellular trafficking, and controllable release of loaded therapeutic cargoes.

Extracellular vesicles (EVs), nanoscale lipid bilayer vesicles, secreted through the endosomal-multivesicular body (MVB) biogenesis pathway, have garnered extensive research interest. EVs carry abundant proteins, lipids, coding and non-coding RNAs, and other bioactive cargoes, and mediate intercellular signal communication in diverse physiological and pathological processes [5]. Derived from living cells, EVs exhibit favorable intrinsic biocompatibility, complete biodegradability, and low inherent immunogenicity. Membrane surface proteins of EVs mediate specific cellular recognition and endocytic uptake, while the lipid bilayer shell can protect internal bioactive cargoes from degradation by circulating nucleases and proteases. In addition, EVs derived from specific cell types possess inherent tissue-homing capacity, such as tropism toward inflammatory lesions and homologous tumor cell recognition [6]. Accordingly, EVs can serve as delivery vehicles for small chemotherapeutic molecules, functional proteins, siRNA, miRNA, mRNA, and multiple other therapeutic cargoes, showing broad application prospects in chemotherapy, gene therapy, and tumor immunomodulatory treatment [7].

Nevertheless, natural EVs lack satisfactory tumor-targeting performance. First, their systemic biodistribution and cellular uptake are dominated by the intrinsic biological properties of parent cells, making it difficult to achieve highly selective recognition of specific malignant cell subsets. Second, cargo loading efficiency, intracellular release efficiency, and batch-to-batch preparation consistency of natural EVs require further optimization. Third, the in vivo biological behaviors of natural EVs are susceptible to plasma protein adsorption, hepatic and splenic clearance, administration routes, and tumor heterogeneity. Notably, EVs secreted by tumor cells may carry oncogenic proteins, tumor-promoting microRNAs, and immunosuppressive membrane molecules, which bring potential risks of accelerating tumor progression [8]. Furthermore, standardized protocols for EV isolation, large-scale mass production, long-term storage stability, and unified quality control systems have not yet been fully established [9]. All the above limitations hinder the clinical translational application of natural EVs as tumor therapeutic carriers.

In recent years, researchers have realized EV functionalization *via* multiple strategies, including biological genetic engineering, chemical covalent conjugation, noncovalent surface anchoring, and hybrid membrane fusion. Targeting ligands, recognition-responsive functional motifs, and therapeutic cargo modules are anchored onto EV membranes or encapsulated inside vesicles to enhance their tumor tissue accumulation, specific cellular recognition, and intracellular cargo release capacity. Functionalized EVs can recognize tumor-overexpressed surface receptors *via* modified targeting peptides, single-chain antibodies, nanobodies, aptamers, or small-molecule ligands [6]. Meanwhile, EVs can be engineered to respond to abnormal biochemical signals within the tumor microenvironment, such as extracellular acidification, hypoxia, overexpressed enzymes, and redox imbalance, to realize lesion-triggered cargo release or surface ligand exposure.

To exert antitumor function, EVs must maintain structural stability during systemic circulation and successfully reach tumor lesions. After arriving at tumor tissue, EVs need to respond to local microenvironmental signals and bind specific receptors on tumor cell membranes. Following endocytic internalization, loaded therapeutic cargoes must escape endosomal and lysosomal sequestration to exert pharmacological effects in the cytoplasm or specific subcellular organelles [2]. Therefore, multi-level functional modification of EVs is required to realize sequential targeting toward tumor tissues, tumor cell membranes, and intracellular compartments, achieving synergistic, precise tumor killing across the whole delivery cascade.

Based on the above background, this review summarizes recent advances in functionalized EVs for tumor-targeted therapy from two interrelated dimensions: EV modification strategies and precision delivery strategies. We first introduce the classification, biological composition, inherent advantages and limitations of EVs as tumor delivery vehicles, and summarize mainstream EV isolation, characterization, and standardized quality control criteria. Next, we classify and elaborate four major modification strategies, biological genetic engineering, chemical covalent surface conjugation, noncovalent membrane anchoring and hybrid membrane fusion, and analyze the construction methods of modified targeting ligands, stimulus-responsive structural motifs, and loaded therapeutic cargoes. Subsequently, based on the different endpoints of precise delivery mediated by functionalized EVs, they can be classified into tumor microenvironment delivery, intracellular delivery into tumor cells, and dual delivery to both the tumor microenvironment and the tumor cell.

Unlike existing review articles classified by modification materials, preparation technologies, or loaded therapeutic cargoes, this review focuses on the sequential action sites and synergistic relationships of diverse functional modules throughout the entire tumor delivery process.

## 2. Construction Strategies of Functionalized EVs

### 2.1. Sources, Composition and Preparation of EVs

#### 2.1.1. Sources of EVs

EVs are nanoscale lipid bilayer vesicles secreted by living cells *via* the endosomal-multivesicular body biogenesis pathway, loaded with diverse nucleic acids, proteins, and lipids to mediate intercellular communication in various physiological and pathological processes [10]. According to preparation sources, EVs can be divided into three major categories: animal-derived EVs, plant-derived EVs and microorganism-derived EVs.

Multiple cell types are capable of secreting EVs, including T lymphocytes, B lymphocytes, macrophages, dendritic cells, natural killer (NK) cells, mesenchymal stem cells (MSCs), vascular endothelial cells, and malignant tumor cells [11]. Among these, macrophages, dendritic cells, and MSCs are the most extensively utilized donor cells in tumor delivery research. Donor cell lineage, activation status, and in vitro culture conditions collectively determine the membrane protein, lipid, and nucleic acid cargo profiles of secreted EVs, which further modulate them in vivo distribution, cellular uptake, and ultimate therapeutic efficacy. Therefore, rational selection of donor cells is an essential first step in constructing functionalized EV-based delivery systems. Furthermore, EVs are widely distributed in various body fluids, including plasma, serum, saliva, urine, peritoneal ascites, milk, bile, and cerebrospinal fluid. Animal-derived EVs present in body fluids can reflect the physiological and pathological states of the organism or specific tissues and are predominantly used for biomarker screening and dynamic disease monitoring. However, animal-derived EVs exhibit pronounced heterogeneity in origin and are prone to contamination by lipoprotein particles, protein aggregates, and heterogeneous EV subpopulations. When utilized as therapeutic carriers, standardized manufacturing and quality control remain considerable challenges.

Plant-derived EVs can be extracted from diverse raw materials, including fresh fruits and leaves, rhizomes and tubers, plant suspension cell cultures, and plant tissue secretions [12]. As novel natural therapeutic nanocarriers, plant-derived EVs possess excellent biocompatibility and low immunogenicity. Nevertheless, broad plant sources lead to prominent interspecies heterogeneity, and their inherent weak tumor-targeting capacity causes short in vivo circulation half-life and limited antitumor efficacy, severely restricting their further application in tumor nanomedicine [13].

Microorganism-derived EVs can be isolated from bacterial and fungal cultures or from body fluids such as blood and urine of infected hosts [14]. As novel natural nanocarriers, microorganism-derived vesicles exhibit high stability and can carry diverse bioactive cargoes, including toxins, adhesins, immunomodulatory factors, and nucleic acids, and are involved in microbe–host interactions, biofilm formation, and immune evasion. However, microorganism-derived vesicles display pronounced heterogeneity, as both pathogenic and nonpathogenic microorganisms can release them, and specific biomarkers remain limited. Consequently, isolation, purification, and quantitative standardization still face considerable challenges, severely restricting their further application in the diagnosis and treatment of infectious diseases.

Overall, the source of EVs determines their membrane surface molecules, endogenous cargo components, and inherent biological functions. For tumor delivery research, EV source selection should comprehensively consider preparation yield, raw material accessibility, natural tissue-homing tropism, immunogenicity, endogenous cargo composition, culture reproducibility, and potential tumor-promoting risks rather than merely focusing on a single indicator.

#### 2.1.2. Composition and Biological Characteristics of EVs

EV membrane surfaces are highly enriched with marker proteins, including CD9, CD63, CD81, Lamp2b, integrins, cell adhesion molecules, heat shock proteins, and other donor cell-derived membrane proteins. These components participate in EV biogenesis, circulatory structural stability, cell recognition and endocytic uptake, and can serve as natural membrane anchoring sites for subsequent surface functional modification [15]. The interior lumen of EVs encapsulates diverse bioactive substances, including mRNA, miRNA, lncRNA, fragmented genomic DNA, metabolic enzymes, and transcription factors. In addition, EVs derived from distinct donor cells inherit partial biological characteristics of parent cells and carry unique cargo subsets [16]. For instance, EVs secreted by immune cells exhibit inherent tropism toward inflammatory tissues, while tumor cell-derived EVs realize homologous tumor cell recognition *via* matched membrane adhesion proteins. These natural biological features lay the foundation for EV-based tumor delivery systems; however, in vivo performance is easily disturbed by preparation protocols, administration routes, and tumor heterogeneity, and cannot be regarded as stable and reproducible active tumor-targeting capacity.

The lipid bilayer shell of EVs can effectively protect internal nucleic acids and proteins from degradation by circulating hydrolases, improving the chemical stability of encapsulated cargoes in body fluid environments [17]. EVs can be internalized by recipient cells through multiple pathways, including membrane fusion, receptor-mediated endocytosis, and macropinocytosis, to deliver loaded bioactive substances into target cells. All the above structural and functional properties constitute the core basis for EVs to act as natural biological delivery carriers.

#### 2.1.3. Isolation and Characterization of EVs

Efficient isolation and purification of EVs are fundamental prerequisites for subsequent structural characterization, biological function evaluation, and therapeutic safety verification [18]. Biological raw samples usually contain cell debris, protein aggregates, lipoprotein particles, and heterogeneous EV subsets simultaneously. A single isolation technology cannot simultaneously realize high preparation purity, high recovery yield, and complete vesicle structural integrity. Therefore, isolation schemes need to be selected according to sample sources, sample volume, experimental purposes, and follow-up applications; combined multi-step purification is recommended when high-purity EV preparation is required.

Mainstream EV isolation technologies include size-exclusion chromatography (SEC) [19,20,21,22], tangential flow ultrafiltration (TFF) [23,24,25,26], immunoaffinity-based capture [27,28,29], differential ultracentrifugation [30,31], density gradient ultracentrifugation [32,33,34] and polymer co-precipitation [35,36]. Differential ultracentrifugation is suitable for large-volume sample processing, but suffers from long operation duration, limited preparation purity, and risks of vesicle aggregation and membrane structural damage. Density gradient ultracentrifugation improves sample purity and enables separation of heterogeneous vesicle subsets, yet the operation process is complicated and time-consuming. Polymer co-precipitation features simple operation and high recovery yield but easily causes co-precipitation of non-vesicular impurities and residual polymer contaminants. SEC maintains mild separation conditions with minimal damage to vesicle structure and favorable experimental reproducibility, making it suitable for complex samples such as plasma and serum; however, its single-batch sample loading capacity is limited, so it is often combined with ultrafiltration for pre-concentration. TFF is applicable for concentration and preliminary separation of large-volume raw samples, but its separation efficiency is restricted by membrane protein adsorption, membrane pore clogging, and fluid shear force. Immunoaffinity-based capture can specifically enrich vesicle subsets expressing designated membrane markers, yet this technology has high preparation costs, low single-batch throughput, and risks of selective loss of EVs subsets due to heterogeneous marker expression levels.

After EV isolation, comprehensive characterization should not rely solely on particle size distribution or single positive marker detection. Multidimensional evaluation covering vesicle morphology, particle size distribution, particle concentration, canonical membrane protein markers, intracellular origin-specific proteins, and non-vesicular impurity markers is mandatory. Common characterization techniques include transmission electron microscopy (TEM) or cryogenic electron microscopy, nanoparticle tracking analysis (NTA), dynamic light scattering (DLS), western blotting, and flow cytometry. For functionalized modified EVs, additional characterization items are required: surface modification ligand abundance, in vitro structural stability, cargo loading efficiency, free cargo residual ratio, cargo release kinetics, and comparative analysis of vesicle structure and biological activity before and after modification. Only after complete verification of vesicle identity, preparation purity, structural integrity, and core quality indicators can comparable and interpretable experimental results be achieved through subsequent surface modification and tumor-targeting functional evaluation.

### 2.2. Therapeutic Functions of Natural EVs

#### 2.2.1. Therapeutic Effects Mediated by Endogenous EV Cargoes

The inherent antitumor activity of natural EVs originates from their endogenous bioactive cargoes [37], and the typical components are summarized in Table 1. For example, Kim et al. prepared Lithospermum erythrorhizon callus-derived extracellular vesicle-like nanovesicles (LELVs). Endogenous shikonin and its derivatives loaded in LELVs significantly promote proliferation and migration of normal human dermal fibroblasts (NHDFs), and upregulate type I collagen synthesis. In a lipopolysaccharide (LPS)-induced inflammatory skin wound mouse model, LELV treatment accelerated epidermal re-epithelialization, reduced local inflammatory cell infiltration, and improved tissue remodeling; histological staining confirmed elevated collagen deposition and a relieved inflammatory response [38]. Yang et al. extracted unmodified turmeric-derived extracellular vesicles (TEVs). Endogenously carried curcumin within TEVs inhibited the Wnt/β-catenin signaling pathway, thereby enhancing immune infiltration by 6.1-fold. In a lung cancer mouse model, the combination achieved 64% primary tumor suppression and 81% metastasis reduction compared with monotherapy, demonstrating full-cycle synergistic immunomodulation [39].

The endogenous biological activity of natural EVs is strictly dependent on donor cell sources and treatment microenvironments. Identical parent cells cultured under different stimulation conditions or medium formulations can secrete EVs with drastically different cargo profiles and biological functions. Therefore, relying solely on the endogenous cargoes of unmodified EVs for disease treatment leads to unstable therapeutic efficacy, which cannot meet the requirements of precision tumor therapy. To break through this limitation, exogenous therapeutic cargoes can be loaded into natural EVs to enhance their tumor-targeting performance and antitumor efficacy.

#### 2.2.2. Exogenous Cargo Loading for Enhanced Therapeutic Delivery

EVs can encapsulate diverse exogenous therapeutic agents, including chemotherapeutic small molecules (doxorubicin, paclitaxel, docetaxel) and biological macromolecules (siRNA, miRNA, mRNA, functional proteins, and gene editing-related components). Encapsulation inside EV lipid shells can reduce cargo degradation during systemic circulation, improve cellular uptake and intracellular utilization efficiency, and potentially mitigate nonspecific distribution and systemic organ toxicity induced by free therapeutic agents.

EV cargo loading technologies are divided into two major categories: pre-secretion endogenous loading and post-isolation exogenous loading. Pre-secretion endogenous loading refers to genetically engineering donor cells to produce therapeutic nucleic acids or proteins encapsulated inside EVs during their biogenesis. For instance, Yim et al. constructed an EXPLOR delivery platform based on blue light-triggered reversible protein–protein interaction between CRY2 and CIBN in HEK293T cells, realizing active loading of soluble functional proteins (Cre recombinase as a model cargo) into newly secreted EVs. This pre-secretion loading strategy achieves efficient cytoplasmic delivery of functional proteins into target recipient cells [48]. Post-isolation exogenous loading utilizes co-incubation, electroporation, sonication, mechanical extrusion, or repeated freeze–thaw cycles to load therapeutic small molecules or nucleic acids into purified isolated EVs. As a representative example, Alvarez-Erviti et al. extracted dendritic cell-derived EVs and loaded BACE1-targeted siRNA *via* post-isolation electroporation. Combined with RVG peptide surface modification, this engineered EV system realizes blood–brain barrier penetration and brain-specific targeted delivery, achieving efficient in vivo silencing of target gene expression [49]. The two loading strategies exhibit distinct advantages and limitations in loading efficiency, cargo structural stability, EV membrane integrity damage, and large-scale production scalability.

Although exogenous cargo loading partially compensates for the functional defects of natural EVs, current loading technologies still face multiple technical bottlenecks. Pre-secretion endogenous loading requires complex genetic modification of donor cells with long experimental cycles; loading efficiency and available cargo types are restricted by the inherent biosynthetic capacity of parent cells [50]. Post-isolation exogenous loading features flexible operation, yet physical loading methods (sonication, electroporation) may disrupt EV membrane integrity, destroy natural tumor-homing capacity, or trigger cargo leakage. Simple passive co-incubation only achieves extremely low loading efficiency, especially for macromolecular protein and nucleic acid therapeutics [51]. Furthermore, natural EVs lack precise tissue-targeting capacity, and their random systemic distribution severely restricts the progress of clinical translational research [52]. To resolve these drawbacks, multidimensional surface functional modification of EVs is required to improve their circulatory stability, tumor-targeting specificity, and antitumor therapeutic efficacy.

### 2.3. Functional Modification Strategies for EVs

#### 2.3.1. Bioengineering of EV-Secreted Parent Cells

Genetic engineering of parent cells prior to EV secretion is a mainstream strategy to produce inherently functionalized EVs (Figure 1). This biological modification method fuses the gene sequence of targeting functional modules (targeting peptides, antibody fragments, receptor ligands, etc.) with the coding sequence of selected EV membrane marker proteins. After transfection into donor cells, secreted EVs naturally display targeting molecules on their membrane surface. For example, Cho et al. genetically engineered donor cells in 2023 to drive co-expression of anti-CD19 single-chain variable fragment (aCD19 scFv) and PD-1 protein on secreted EVs, constructing bispecific engineered EVs (BsEVs). The aCD19 scFv guided specific targeting toward solid tumor cells overexpressing huCD19, while surface PD-1 protein blocked tumor-expressed PD-L1 to relieve local immunosuppression. Moreover, these bispecific EVs captured circulating tumor-derived EVs to inhibit the construction of pre-metastatic niches in distant organs [53]. Bioengineering modification of donor cells offers advantages in that the modification is relatively stable, causes minimal damage to membrane structures, and is suitable for sustained, specific functional expression. Its drawbacks include complex cell transfection or gene editing workflows; modification efficiency that is influenced by cell type and expression level; and the potential to alter the biological characteristics of donor cells as well as the endogenous components of exosomes. Therefore, safety and batch consistency evaluations associated with genetic manipulation need to be carefully considered [50].

#### 2.3.2. Direct Surface Modification of Purified EVs

##### Chemical Covalent Modification

Chemical covalent modification is conducted after EV purification. This strategy leverages abundant amino, carboxyl, and sulfhydryl functional groups on EV membrane surfaces to covalently anchor targeting peptides, antibodies, aptamers, fluorescent tracers, and stimulus-responsive structural motifs *via* specific chemical reactions (Figure 1). Widely adopted reaction systems include EDC/NHS-mediated amide bond formation, maleimide-sulfhydryl coupling, and bio-orthogonal click chemistry. Tang et al. covalently conjugated the HA1 aptamer onto grapefruit-derived vesicle-like nanovesicles to fabricate an aptamer-functionalized EV. Surface HA1 aptamer modification significantly enhanced specific tumor accumulation and cellular uptake in breast cancer xenograft models, achieving efficient suppression of in vivo tumor proliferation [13]. Smyth et al. pioneered the application of copper-catalyzed azide-alkyne cycloaddition (CuAAC) click chemistry for EV surface functionalization in 2014. This mild aqueous-phase conjugation strategy realized efficient and specific surface modification without altering EV particle size or cellular uptake behavior, verifying that click chemistry reaction conditions caused negligible damage to EV structural integrity and biological functions, laying a solid technical foundation for subsequent biological applications of functionalized EVs [54]. This method offers advantages in that it provides firm binding, makes functional molecules less prone to detachment, and allows high controllability of modification, making it suitable for constructing stable tumor-targeted delivery systems. However, chemical reaction conditions may damage membrane proteins and lipid bilayers; excessive modification can alter exosome particle size, surface charge, uptake pathways, and in vivo distribution. Therefore, optimization is needed to balance coupling efficiency with the preservation of EV biological activity [51].

##### Noncovalent Modification

1. Hydrophobic Membrane Insertion

Hydrophobic insertion relies on spontaneous insertion of hydrophobic lipid tails (DSPE, cholesterol derivatives) into the phospholipid bilayer of EV membranes, displaying conjugated targeting ligands or PEG shielding chains on the EV outer surface. Driven by hydrophobic intermolecular forces, this strategy maintains intact EV membrane structure without destructive chemical reactions. For example, Jang et al. co-incubated bovine milk-derived exosomes (m Exo) with folate (FA)-conjugated 16:0 PE lipid (PE-FA); the hydrophobic lipid tail of PE spontaneously inserts into the m Exo phospholipid bilayer *via* post-insertion, exposing the hydrophilic FA targeting ligand on the outer membrane surface. They constructed an FA-modified m Exo-FA delivery system, loaded doxorubicin (Dox) to form Dox @m Exo-FA, which enhanced targeted uptake by FR-high HCT116 colorectal cancer cells through FA receptor-mediated endocytosis. In HCT116 tumor-bearing mice, Dox @mExo-FA significantly inhibited tumor growth while reducing free Dox-induced body weight loss, splenic contraction, hepatotoxicity, and cardiotoxicity [55]. Zou et al. mixed diacyl lipid-aptamer conjugates with purified EVs in 2019. The lipid hydrophobic tails inserted into EV membranes, displaying surface-exposed targeting aptamers to construct aptamer-functionalized EVs (Apt-EVs). This modification endows EVs with specific recognition capacity toward malignant tumor cells, realizing efficient targeted delivery of small-molecule chemotherapeutics and fluorescent imaging probes [56]. Hydrophobic insertion is mild and simple, suitable for anchoring lipid ligands or functional molecules to the membrane, but molecular detachment or lipid exchange may occur in the circulation or complex body fluids.

2. Electrostatic Adsorption Modification

EV membrane surfaces carry inherent negative electrostatic charge, which enables adsorption of positively charged functional molecules (cationic lipids, cationic polymers) *via* electrostatic interaction to realize simple surface modification. Tamura et al. mixed positively charged cationized pullulan with negatively charged mesenchymal stem cell-derived EVs to complete surface functionalization *via* electrostatic adsorption. Modified EVs exhibited drastically enhanced internalization efficiency in HepG2 hepatocellular carcinoma cells mediated by cell-surface asialoglycoprotein receptors. In a ConA-induced acute liver injury mouse model, cationized pullulan-modified EVs achieved significantly elevated liver-specific accumulation, effectively reducing plasma ALT levels, shrinking hepatic necrotic areas, and regulating the expression of pro-inflammatory cytokines [57]. Electrostatic adsorption requires no complex chemical reactions and is suitable for the rapid loading of nucleic acids, proteins, and charged nanomaterials, but binding stability is relatively weak. Additionally, altered surface charge may affect extracellular vesicle stability and nonspecific uptake.

3. Ligand–Receptor Specific Binding Modification

This modification strategy utilizes highly specific binding affinity between ligands and membrane receptors to anchor functional molecules onto intrinsic surface proteins of EVs. Li et al. constructed iRGD peptide-engineered EVs (iRGD-EVs) and loaded siMYC *via* electroporation to fabricate a targeted triple-negative breast cancer delivery system. Surface iRGD peptide specifically binds integrin αvβ3, which is highly expressed on triple-negative breast cancer cell membranes, improving EV-specific uptake efficiency by 3.8-fold relative to unmodified control groups. In vitro cell experiments verified that iRGD-EV-siMYC significantly suppressed triple-negative breast cancer cell proliferation and induced cell apoptosis; obvious apoptotic cell death was also observed in patient-derived tumor organoid (PDO) models. In Hs578T xenograft tumor-bearing mice, iRGD-EV-siMYC displayed excellent tumor-targeting capacity, achieving a tumor volume inhibition rate up to 76.4% [58]. Ligand–receptor affinity modification offers good selectivity and mildness, but its efficacy depends on the affinity of the binding pair, the abundance of extracellular vesicle surface markers, and competitive binding in vivo; consistency during large-scale preparation requires further verification.

##### Hybrid Membrane Fusion Modification

Membrane fusion mixes purified EVs with liposomes, cell membrane vesicles, or other membranous materials *via* co-incubation, repeated freeze–thaw cycles, mechanical extrusion, or electrofusion to generate hybrid vesicle delivery systems. Sato et al. adopted repeated freeze–thaw treatment to induce membrane fusion between EVs and synthetic liposomes to prepare hybrid EV–liposome nanocarriers. This method integrated functional lipids or membrane components from liposomes into EV lipid bilayers, enabling tunable regulation of EV–cell interaction and endocytic uptake behavior *via* adjusting membrane lipid composition [59]. This approach combines the biological characteristics of EVs with the designability of artificial liposomes, enhances drug-loading capacit, and alters cellular uptake and targeting behaviors by adjusting lipid composition, surface ligands, or membrane proteins. However, this method may lead to heterogeneity in the fusion products, increased particle size, and rearrangement of the membrane structure. Simultaneously, fusion efficiency, the degree of retention of natural EV functions, and impurity removal all affect the quality of the final preparation.

As summarized in Table 2, *via* various modification strategies, functionalized EVs exhibit remarkable therapeutic efficacy in the treatment of diverse diseases, presenting promising application prospects.

## 3. Precision Delivery of Functionalized EVs in Antitumor Therapy

### 3.1. Tumor Microenvironment-Targeted Precision Delivery

Solid tumors possess unique biochemical characteristics distinct from healthy tissues, including extracellular acidosis, hypoxia, aberrant enzymatic activity, redox imbalance, and dense extracellular matrix deposition [71,72,73]. These intrinsic differences lay the foundation for designing precision delivery in the TME. Such systems remain structurally stable during systemic circulation and within normal tissues, yet undergo cleavage of sensitive chemical linkers, shedding of protective shielding layers, exposure of hidden targeting ligands, carrier disassembly, or controlled release of therapeutic cargoes upon arrival at tumor lesions (Figure 2). For instance, Zhang et al. developed a milk-derived EV delivery platform (Exo@Dox-EPT1), where doxorubicin (DOX) was conjugated to EV surfaces via acid-cleavable bonds. Upon exposure to the acidic tumor microenvironment, these linkers hydrolyzed to release free DOX, enabling site-specific chemotherapy against oral squamous cell carcinoma [74]. The extracellular matrix of solid tumors maintains a markedly acidic microenvironment relative to normal tissues. To exploit this trait, pH-labile chemical linkages, charge-reversal polymers, or acid-sensitive structural moieties can be engineered onto EV surfaces or incorporated into composite delivery platforms. These constructs retain structural integrity under physiological neutral pH, while triggering protective layer detachment, ligand unmasking, carrier dissociation, or payload release within acidic tumor niches. This design mitigates premature cargo leakage during circulation and facilitates deeper tumor penetration as well as enhanced cellular internalization [75,76].

In addition to the aforementioned markedly acidic tumor microenvironment, solid tumors overexpress abundant matrix metalloproteinases, cathepsins, and hyaluronidases, which can be harnessed to activate functionalized EVs in situ. Enzyme-cleavable peptide substrates, degradable protective coatings, or hydrolyzable steric shielding layers can be integrated into EV carriers. This design minimizes nonspecific ligand exposure during systemic circulation; once reaching tumor lesions, enzymatic hydrolysis triggers ligand exposure, improved tissue penetration, or rapid intracellular cargo release [77,78]. Hong et al. constructed PH20 hyaluronidase-loaded engineered EVs (Exo-PH20). Surface-displayed PH20 degraded hyaluronic acid, a major component of the dense tumor extracellular matrix, thereby eliminating diffusion barriers and enabling deeper penetration of EVs and loaded therapeutics into tumor parenchyma. Meanwhile, hyaluronic acid degradation remodeled the tumor immune microenvironment to boosted intratumoral immune cell infiltration [79].

### 3.2. Tumor Cell Intracellular Precision Delivery

According to different designs of the delivery recognition targets, tumor cell intracellular precision delivery can be further classified into tumor cell membrane single-recognition delivery, tumor cell intracellular single-recognition delivery, tumor microenvironment–tumor cell membrane dual-recognition delivery, tumor microenvironment–tumor cell intracellular dual-recognition delivery, tumor cell membrane–tumor cell intracellular dual-recognition delivery, and tumor microenvironment–cell membrane–intracellular triple-recognition delivery.

#### 3.2.1. Tumor Cell Membrane Single-Recognition Delivery

Malignant cells aberrantly overexpress multiple surface biomarkers, including HER2, EGFR, integrin αvβ3/αvβ5, CD44, folate receptor α, EpCAM, and transferrin receptor (Table 3). Displaying complementary high-affinity ligands on EV surfaces strengthens specific binding to biomarker-positive tumor cells and promotes receptor-mediated endocytosis [80]. Tian et al. genetically fused the αv integrin-targeting iRGD peptide (CRGDKGPDC) with the EV membrane marker Lamp2b, then transfected this fusion construct into immature mouse dendritic cells. Secreted EVs naturally displayed surface iRGD peptides, and purified EVs were loaded with DOX via electroporation. The iRGD motif specifically bound αv integrin highly expressed on tumor vascular endothelial and cancer cell membranes, enabling active targeted chemotherapy against breast cancer xenografts [81]. In another study, Ohno et al. fused the EGFR-specific GE11 peptide (YHWYGYTPQNVI) to the transmembrane domain of platelet-derived growth factor receptor (PDGFR), followed by transfection into 293T cells to generate GE11-displaying EVs loaded with the tumor-suppressive miRNA let-7a. The GE11 peptide exhibits high binding affinity toward EGFR-overexpressing tumor cells without mitogenic side effects, and the engineered EV system effectively suppressed EGFR-positive breast tumor growth in vivo [82]. Based on the above examples, it can be seen that this single-recognition delivery can enhance tumor cell binding and internalization efficiency, making it particularly suitable for tumor subtypes with defined receptor expression. It also facilitates the construction of theranostic systems combined with imaging probes, chemotherapeutic drugs, or nucleic acid drugs [81]. However, there are also some limitations. For example, target receptors often exhibit inter-patient and intra-tumoral heterogeneity, and some receptors are also expressed in normal tissues. Excessively high ligand modification density may affect extracellular vesicle membrane protein function, and alter in vivo clearance pathways, and steric hindrance can further reduce actual binding efficiency. Therefore, receptor selection should be based on a comprehensive evaluation of expression profiles, normal tissue distribution, and internalization capacity rather than solely on high expression levels in tumor cells.

#### 3.2.2. Tumor Cell Intracellular Single-Recognition Delivery

##### Intracellular Redox-Triggered Release

Tumor cells harbor drastically elevated reactive oxygen species (ROS) and glutathione (GSH) levels compared with healthy somatic cells [86], which serve as endogenous intracellular stimuli to trigger payload release (Table 3). Redox-responsive linkages, including disulfide, diselenide, thioketal, and boronate ester moieties, undergo selective cleavage under distinct redox conditions, thereby triggering the release of small-molecule drugs or nucleic acid therapeutics from EV-based composite carriers. Specifically, disulfide and diselenide linkers are typically cleaved under reducing conditions, particularly in GSH-rich environments, whereas thioketal and boronate-containing systems generally respond to ROS [87]. Wu et al. developed a macrophage EV-camouflaged degradable silica nanoplatform (CSI@Ex-A) co-loaded with catalase (CAT) and sonosensitizer ICG, with surface AS1411 aptamer modification for glioma targeting. After internalization by glioma cells, high intracellular GSH induces degradation of the silica framework to release CAT, which catalyzes H_2_O_2_ decomposition into oxygen to alleviate intratumoral hypoxia. The combination of GSH depletion and in situ oxygen supply synergistically amplifies sonodynamic therapeutic efficacy [88]. Xu et al. constructed ROS-responsive liposomal nanocomplexes cloaked with macrophage-derived EV membranes (E-Lip-DTX/si), co-encapsulating docetaxel (DTX) and Bcl-2-targeted siRNA within liposomal cores. Leveraging the inherent tumor-homing capacity of macrophage EVs, the nanocomplexes selectively target breast cancer cells. Elevated intracellular ROS cleaved redox-sensitive linkers inside liposomes post-internalization, simultaneously releasing DTX and siRNA to realized synergistic chemo- and RNA-interference antitumor therapy [89].

##### Intracellular Enzyme-Triggered Activation

Tumor cells overexpress unique intracellular hydrolases such as cathepsin B, intracellular matrix metalloproteinase fragments, and telomerase, which cleave specific peptide or nucleic acid sequences to activate therapeutic cargoes intracellularly [89]. Jang et al. prepared an oral delivery system (FDX@mEVs) by complexing a cathepsin B-activatable doxorubicin prodrug (FRRG peptide-DOX, FDX) with milk-derived EVs (mEVs). This system relied on the specific binding of mEVs to the intestinal neonatal Fc receptor (FcRn) to mediate transcytosis across the intestinal epithelium, thereby targeting colon tumors. After oral administration, FDX@mEVs traversed the intestinal barrier into the systemic circulation and accumulated in tumor tissues *via* the enhanced permeability and retention (EPR) effect. Following endocytosis by tumor cells, lysosomal cathepsin B recognized and cleaved the FRRG peptide linker, releasing free DOX, which induced tumor cell apoptosis, potently inhibited tumor growth, and simultaneously reduced systemic toxicity [90]. Ahmadi et al. designed a cathepsin B-activatable adenosine-tetrapeptide prodrug (Gly-Phe-Leu-Gly-adenosine) and utilized EVs as a delivery platform. The prodrug was conjugated to adenosine *via* N6-substitution with the tetrapeptide GFLG, which was specifically recognized by cathepsin B overexpressed in tumor cells. EVs were derived from AsPC-1 pancreatic cancer cells and RAW 264.7 macrophages, and the prodrug was loaded by sonication. After internalization by AsPC-1 pancreatic cancer cells, lysosomal cathepsin B cleaved the GFLG sequence, releasing active adenosine, inhibiting tumor cell proliferation, and inducing apoptosis. In vitro cell experiments confirmed that the prodrug-loaded EVs exhibited stronger cytotoxicity and pro-apoptotic activity than the free prodrug, with lower toxicity to normal L929 cells. Molecular docking simulation verified the high affinity of the prodrug for the active site of cathepsin B [91].

##### Subcellular Organelle-Targeted Delivery

When therapeutic agents exert pharmacological functions within specialized subcellular compartments (nucleus, mitochondria, lysosomes, endoplasmic reticulum), organelle-localized targeting modules can be modified onto EV surfaces to regulate their intracellular trafficking and distribution [92]. Nguyen Cao et al. constructed TPP-modified sonosensitizer-loaded EVs (T-Ce6-EVs) that accumulate selectively in mitochondria *via* cationic TPP moieties. Upon ultrasonic irradiation, mitochondrially enriched Ce6 generated massive reactive oxygen species to disrupted mitochondrial function and accelerated tumor cell apoptosis [93]. Fan et al. engineered chimeric peptide-functionalized EVs (ChiP-Exo). The chimeric peptide integrates plasma membrane-targeting sequences, photochemical internalization (PCI) motifs, and nuclear localization signals (NLSs), which were anchored onto EV surfaces *via* hydrophobic lipid insertion, with photosensitizers encapsulated inside the EV lumen. This system adopted a two-step light irradiation protocol: the first light stimulus generated plasma membrane-localized ROS to induced mild membrane damage and promoted lysosomal escape; guided by NLS sequences, residual carriers accumulated within cell nuclei, where secondary irradiation triggered an intranuclear ROS burst to eliminated tumor cells. Sequential dual-target phototherapy achieved remarkable tumor growth inhibition with negligible systemic organ toxicity [94].

#### 3.2.3. Tumor Microenvironment–Tumor Cell Membrane Dual-Recognition Delivery

The core design principle of integrating tumor microenvironment responsiveness with cell-surface active targeting is as follows (Table 4). First, intrinsic TME cues (extracellular pH, elevated ROS, overexpressed enzymes, hypoxia) trigger shedding of surface protective layers or unmasking of hidden targeting ligands. Next, the exposed ligands (peptides, antibodies, aptamers, or small molecules) specifically bind receptors on malignant cells, triggering cargo release either extracellularly or intracellularly to eliminate tumor cells.

Nie et al. developed a pH-sensitive EV nanoconjugate that integrates both TME responsiveness and cell membrane recognition. Anti-CD47 and anti-SIRPα antibodies were anchored onto M1 macrophage-derived EVs *via* acid-cleavable linkers. Once exposed to the acidic tumor niche, the linkers hydrolyzed to release free antibodies, which separately bound CD47 on tumor cells and SIRPα on macrophages. This blocked the “don’t eat me” immune checkpoint signal and reinforced macrophage-mediated phagocytosis of tumor cells [95].

Zheng et al. fabricated a dual-nanobody functionalized milk-derived EV nanomedicine termed 7D12/KN035-iEV. Two nanobodies (EGFR-targeting 7D12 and PD-L1-targeting KN035) were site-specifically conjugated to EV surfaces *via* enzymatic ligation, while miR-21 inhibitor was encapsulated within the EV lumen. The dual-targeting platform precisely guided EVs toward EGFR-positive triple-negative breast cancer (TNBC) cells and PD-L1-expressing tumor-associated macrophages (TAMs). Delivered miR-21 inhibitor remodeled the suppressive tumor immune microenvironment. In TNBC mouse models, this design markedly restrained primary tumor proliferation and pulmonary metastasis, facilitated polarization of antitumor M1 macrophages, and elevated intratumoral infiltration of cytotoxic CD8^+^ T cells [85].

#### 3.2.4. Tumor Microenvironment–Tumor Cell Intracellular Dual-Recognition Delivery

This dual-modal targeting strategy, similar to the TME-cell membrane combined strategy, has exhibited significant antitumor efficacy (Table 4). It enables spatiotemporally coordinated delivery with a sequential cascade: TME-triggered priming followed by intracellular site-specific cargo release, ensuring therapeutic agents exert precise cytotoxicity inside tumor cells. Unique biochemical signals within the tumor microenvironment act as primary switches to initiate EV tumor accumulation, unmask surface functional motifs, or trigger preliminary cargo release. After cellular internalization, intracellular stimuli (high GSH levels, acidic lysosomal pH, ATP deficiency) further drive complete liberation or activation of loaded therapeutics. Zhao et al. loaded porphyrin-conjugated spider peptide P1 into glioma cell-derived EVs (EVs@P1). The system relies on inherent homologous tumor tropism of glioma EVs to facilitate cellular uptake. Following endocytosis, P1 was hydrolyzed under acidic lysosomal conditions to expose mitochondrial-targeting sequences. Upon ultrasonic irradiation, the liberated peptide self-assembled into mitochondrial-localized nanofibers that generated abundant reactive oxygen species (ROS), realizing synergistic antiglioma therapy combining homologous uptake and mitochondrial-specific oxidative damage [96].

Wang et al. constructed an endoplasmic reticulum (ER)-homing milk EV delivery system named CEL/siPD-L1@Exo-KDEL. Both celastrol (CEL) and PD-L1-targeting siRNA were encapsulated inside milk EVs, whose surfaces were functionalized with ER-specific KDEL peptides. Guided by KDEL, EVs selectively trafficked to the ER of tumor cells. Intracellularly delivered CEL induced immunogenic cell death (ICD), promoted the secretion of damage-associated molecular patterns (DAMPs), and accelerated dendritic cell maturation. Meanwhile, co-delivered siRNA suppressed surface PD-L1 expression on malignant cells. Collectively, the two payloads synergistically amplified intratumoral CD8^+^ T cell recruitment and activation, resulting in robust suppression of orthotopic colorectal tumor growth and potent systemic antitumor immune responses [97].

#### 3.2.5. Tumor Cell Membrane–Tumor Cell Intracellular Dual-Recognition Delivery

This dual-target strategy realizes multi-layered specific recognition of malignant cells. By sequentially identifying cell-surface receptors followed by intracellular stimulus signals, the delivery system minimizes off-target damage to healthy cells at the single-cell level. Highly expressed tumor membrane receptors act as specific molecular address tags. Functionalized EVs carry surface-targeting motifs to selectively bind and internalize into cancer cells. Once inside tumor cells, intracellular biochemical cues trigger controlled liberation or activation of therapeutic payloads, fulfilling a sequential delivery cascade: specific cell recognition, cellular uptake, compartment-specific cargo release (Table 5).

Zhan et al. modified blood-derived EV membranes with magnetic moieties and endocytic L17E peptides *via* noncovalent anchoring, and co-encapsulated DOX alongside a cholesterol-conjugated miR-21 inhibitor. After L17E-mediated tumor cell endocytosis, DOX translocated to the cell nucleus to damage genomic DNA, while the miR-21 inhibitor resided in the cytoplasm to exert gene-silencing effects. This platform integrates cell-surface uptake with multi-compartment intracellular antitumor therapy [98].

Yueh et al. utilized the PiggyBac transposon system to co-express miR-124 and PD-1 in human umbilical cord mesenchymal stem cells (hUC-MSCs), generating engineered hUC-MSC/miR-124-PD-1 cells and their secreted EVs. Leveraging the intrinsic glioma-homing tropism of hUC-MSC-derived EVs, the system targeted orthotopic glioblastoma lesions. Surface PD-1 protein binded tumor-expressed PD-L1 to reactivate antitumor T cell immunity, whereas encapsulated miR-124 silenced CDK4/CDK6 to trigger tumor cell cycle arrest and apoptotic death. In orthotopic glioblastoma mouse models, these engineered EVs selectively eliminated glioma cells, extended animal survival duration, and remodeled the suppressive tumor immune microenvironment—they boosted intratumoral infiltration of CD8^+^ T cells, dendritic cells (DCs), and M1 macrophages while repressing regulatory T cells (Tregs) and myeloid-derived suppressor cells (MDSCs)—without observable systemic toxic side effects [99].

**Table 5:** Representative functionalized EVs and their therapeutic efficacy based on tumor cell membrane–intracellular dual-recognition precision delivery strategies.

EV Source	Modifying Component	Model	Therapeutic Efficacy	Ref.
HEK293T	iRGD peptide & miR-150-3p	Melanoma	Inhibit primary tumor growth; inhibit lung metastasis; enhance CD8^+^ T infiltration; synergize with ICI	[68]
Whole blood	Magnetic molecules & L17E endocytic peptide	Multiple solid tumors	Low toxicity; marked tumor suppression	[98]
HEK293T	iRGD peptide & KRAS siRNA	Lung adenocarcinoma	Downregulate KRAS in vitro; obvious tumor inhibition; induce apoptosis and necrosis	[100]
BM-MSCs, UC-MSCs, ADSCs	Targeting ligands/peptides & tumor suppressive miRNAs	Multiple solid tumors	Inhibit proliferation and migration; induce apoptosis; remodel immune microenvironment; synergistically reverse resistance	[101]
hUC-MSCs	PD 1 protein & miR-124	Glioblastoma	Targeted tumor suppression, tumor volume reduced to ~1/10 of control and prolonged survival; remodel immune microenvironment	[102]

#### 3.2.6. Tumor Microenvironment–Tumor Cell Membrane–Tumor Cell Intracellular Triple-Recognition Delivery

This triple cascade targeting strategy follows a sequential workflow: TME-targeted recognition, tumor cell membrane recognition, and intracellular controlled cargo release. It enables hierarchical discrimination across tissue, cellular, and subcellular scales, thereby minimizing off-target adverse effects after systemic administration (Table 6).

Cheng et al. constructed a multifunctional hybrid EV platform designated Exo@SN38/MnO_2_. In the first cascade stage, loaded MnO_2_ nanoparticles reacted with abundant hydrogen peroxide (H_2_O_2_) accumulated in the tumor microenvironment. Decomposition of MnO_2_ generated oxygen to alleviate intratumoral hypoxia and simultaneously unmasked surface CD47 motifs. Exposed CD47 binded SIRPα on tumor cell membranes to block the “don’t eat me” signal and facilitate EV internalization. Following cellular uptake, the encapsulated chemotherapeutic SN38 translocated into the nucleus to trigger DNA damage, while released Mn^2+^ activated the cytoplasmic cGAS/STING innate immune axis. The synergistic chemoimmunotherapy yielded an 86.4% tumor inhibition rate in CT26 colon tumor-bearing mice, with markedly attenuated injury to healthy tissues relative to untargeted carriers [103].

Guo et al. developed an acid-activatable dynamic hybrid nanoplatform for synergistic triple immunotherapy (photodynamic therapy, ferroptosis, and cGAS–STING pathway activation) against breast cancer. Following intravenous administration, the platform actively targeted and accumulated in the tumor microenvironment *via* the specific affinity of the RS17 peptide for CD47 on tumor cells and the intrinsic tumor-homing capability of M1 macrophage-derived EVs. Upon exposure to the acidic tumor microenvironment, the acid-labile coordination bonds were cleaved, releasing the quenched photosensitizer SR780, Fe^3+^, and REV, thereby achieving targeted drug release at the tumor-cell level. Under 808 nm laser irradiation, the activated SR780 generated abundant reactive oxygen species within tumor cells, inducing immunogenic cell death; the released Fe^3+^ was reduced to Fe^2+^ by intracellular glutathione, triggering Fenton reaction-mediated lipid peroxidation and GPX4 inhibition, leading to ferroptosis; concurrently, REV activated the cGAS–STING pathway, promoting dendritic cell maturation and type I interferon secretion. These three mechanisms synergistically remodelled the immunosuppressive microenvironment and activated CD8^+^ cytotoxic T lymphocytes. In orthotopic 4T1 breast tumor-bearing mouse models, REV@SR780Fe@LEV-RS17 combined with laser irradiation significantly suppressed primary tumor growth with a 79.7% tumor inhibition rate, prolonged median survival to 66.7% at 60 days, and effectively inhibited lung and liver metastases. Compared with free drugs or monotherapy groups, this platform efficiently eradicated tumor cells *via* triple synergistic mechanisms, with no significant histopathological damage to major organs and favorable biosafety [65].

**Table 6:** Representative functionalized EVs based on the triple-recognition delivery strategy and their therapeutic efficacy.

EV Source	Modifying Component	Model	Therapeutic Efficacy	Ref.
M1 macrophages	Acid-responsive deshielding & CD47 targeting & tumor homing & REV & SR780Fe	Breast cancer	Inhibit primary tumor growth, tumor inhibition: 79.7%; suppress distant metastasis, mouse survival rate: 0% to 66.7%; activate potent immune responses	[65]
Exhausted T cells	TME immune neutralization & tumor stemness cell membrane targeting & siTEAD & ROS induced ICD	Triple-negative breast cancer	Suppress tumor stemness, ALDH+ CSCs: 34.3% to 18.2%; effectively inhibit tumor proliferation, pulmonary metastatic nodules: ~35 to ~5; prevent recurrence	[102]
BM-MSCs	CAF reprogramming & integrin α5 targeting & miR-138 & PFD	Pancreatic cancer	Enhance gemcitabine penetration; significantly inhibit primary tumor; suppress peritoneal and liver metastases	[104]
RAW 264.7	pH responsive lysosomal escape & cRGD integrin targeting & 5 FU	Melanoma	Increase tumor accumulation by 2.1-fold;	[105]

### 3.3. Tumor Microenvironment–Tumor Cell Intracellular Dual Delivery

Tumor microenvironment–tumor cell intracellular dual delivery refers to the use of a single delivery system that both relieves immunosuppression and reshapes immunity in the tumor microenvironment and intervenes in key signaling pathways inside tumor cells, thereby producing synergistically enhanced antitumor effects.

Ma et al. developed an engineered EV platform, IDEA, by co-expressing cGAS and VSV-G in producer cells without any scaffold. After intratumoral administration, EVs delivered cGAS into the tumor cell cytoplasm, activating STING/interferon responses, while simultaneously remodeling the TME by promoting antitumoral neutrophil infiltration, activating CD4^+^/CD8^+^ T cells, and converting M2-like to M1-like macrophages. In MC38 tumor-bearing mice, this inhibited tumor growth, prolonged survival, and induced resistance to rechallenge; combined with anti-PD-1, it achieved 100% tumor-free survival. In the poorly immunogenic B16.F10 model, approximately 40% of mice were cured. This dual action—intracellular cGAS delivery to activate STING plus TME remodeling—elicited robust systemic antitumor immunity [106]. Kim et al. developed a folate-modified lipid nano-assembly (FD9R-GW/siIRF3) co-delivering GW4869 and siIRF3. After intratumoral administration, it targeted cancer cells and TAMs via folate receptor. GW4869 inhibited nSMase, suppressing tumor-derived exosome (TEX) release and exosomal PD-L1; siIRF3 silenced IRF3, inhibiting M2-TAM differentiation and CCL22 secretion, reducing Treg recruitment. In 4T1 breast cancer mice, tumor volume decreased by 55%, lung metastasis dropped 4.34-fold, with increased CD8^+^ T cells and M1 macrophages. Combined with anti-PD-1, tumor inhibition was 1.65-fold greater than FD9R-GW/siIRF3 alone, and granzyme B^+^ cytotoxic T cells increased 2.93-fold. This dual action—TEX blockade plus IRF3 silencing—inhibited tumor immune evasion and enhanced antitumor immunity [107].

## 4. Discussion

Functionally modified EVs represent an emerging nanobiotechnology platform demonstrating unprecedented potential in cancer therapy. Through precision engineering strategies, including bioengineering modification, covalent modification, noncovalent modification, and membrane fusion strategies, targeting moieties and therapeutic drugs can be successfully integrated into these natural carriers. These modifications effectively overcome the core challenges of conventional nanomedicines, including poor targeting specificity, high systemic toxicity, and the low biocompatibility of synthetic carriers. In addition to the functional modification of EVs themselves, hybrid functionalization combining EVs with other materials also exists, in which EVs are integrated with synthetic materials to form hybrid functionalized systems. Functional modification of EVs themselves is more suitable for leveraging their natural biocompatibility, low immunogenicity, and intrinsic targeting ability, making them applicable to personalized or precision therapy scenarios; whereas hybrid functionalized systems combining EVs with nanoparticles offer greater advantages in improving drug-loading capacity, enhancing stability, achieving controlled release, and enabling scalable production, making them more suitable for therapeutic needs requiring efficient delivery and batch preparation. The two are not mutually exclusive but represent two selectable strategies for different therapeutic goals and translational stages. Future research needs to further optimize fusion efficiency, establish standardized preparation and quality control processes, and evaluate long-term safety and immune tolerance. In addition, at present, existing reports still lack specific quantitative data on the drug-loading capacity, loading efficiency, and stability of functionally modified extracellular vesicles; future research can be further expanded in this area.

This review focuses on analyzing the strategies by which these functionalized extracellular vesicles achieve precise delivery. According to the different delivery endpoints, they can be classified into tumor microenvironment delivery, intracellular delivery into tumor cells, and tumor microenvironment–tumor cell intracellular dual delivery. Different precise delivery strategies involve different targeting strategies, which can be categorized into tumor microenvironment-responsive, tumor cell membrane-responsive, intracellular-responsive, and multi-target combined responsive systems. Compared with native EVs, these strategies can, to a certain extent, enhance tumor accumulation, cellular uptake, and intracellular release of therapeutics, augment antitumor efficacy, and reduce non-target tissue exposure. Tran et al. were the first to demonstrate in breast cancer that EVs displaying the dual tumor-penetrating peptides iRGD and tLyp1 exhibited superior cellular uptake, drug delivery, and cytotoxicity compared with mono-targeting modifications. In MDA-MB-231 cells, dual-targeting EVs showed approximately 41% higher uptake than WT EVs and were significantly more internalized than single iRGD or tLyp1 EVs. The IC50/GI50 values of dual-targeting Exo-Dox were 3.7–17.0-fold lower than those of free Dox and other EV-based Dox formulations. In MCF-7 cells, the IC50 of dual-targeting Exo-Dox was 6-fold lower than that of free Dox and 3.7-fold lower than that of WT Exo-Dox, whereas in MDA-MB-231 cells it was 4.5-fold lower than free Dox and 3.9-fold lower than WT Exo-Dox. The difference in GI50 was even more pronounced, with the GI50 of dual-targeting EVs being more than 2-fold lower than that of mono-targeting EVs. Single iRGD Exo-Dox reduced IC50/GI50 by only approximately 20–40% compared with WT Exo-Dox, and single tLyp1 was slightly superior to iRGD, but both were inferior to dual-targeting. These data indicate that dual-targeting is not a simple additive effect of mono-targeting but instead produces synergistic enhancement of penetration and therapeutic efficacy, potentially reducing the effective dose and side effects, thereby demonstrating the synergistic advantages of multi-targeting modification [108]. However, their shortcomings are also prominent, including multiple construction steps, difficult quality control, potential interference between modules, and the need for full verification of whether each level of response occurs in vivo as expected. As system complexity increases, the difficulties of scalable preparation, payload homogeneity, long-term stability, and regulatory evaluation increase simultaneously.

In the future, the development focus of functionalized EV targeted delivery should not remain merely on adding targeting ligands or response modules but should shift toward building precision delivery systems with clinical translatability. First, stratified design should be conducted based on receptor expression, degree of hypoxia, acidity level, enzyme profile, and redox characteristics of different tumors, avoiding a “one platform fits all tumors” strategy. Second, a unified in vivo evaluation system should be established to simultaneously compare tumor/normal tissue distribution, cellular uptake, intracellular escape, effective payload release, and therapeutic benefit, rather than evaluating targeting efficacy solely based on in vitro uptake rates. Finally, a promising direction is to adopt moderately complex, mechanism-clear multi-target recognition systems, combined with standardized EV preparation, scalable modification processes, and long-term safety studies, to promote their transition from proof-of-concept to clinical application.

This review summarizes various precise delivery strategies for EVs and, on this basis, outlines the design features of different delivery strategies. To facilitate systematic comparison, Table 7 summarizes the strategies for different delivery endpoints, including tumor microenvironment delivery, intracellular delivery into tumor cells, and tumor microenvironment–tumor cell intracellular dual delivery, from the perspectives of design strategy, clinical translation potential, advantages, and limitations.

## 5. Conclusions and Future Perspectives

Although the existing literature and preclinical evidence indicate that natural and functionalized EVs hold significant promise for disease treatment, developing more versatile and stable modification methods, along with specific precision delivery strategies, is crucial for improving therapeutic efficacy, particularly in tumor-targeted therapy. Currently, while natural EVs possess excellent biocompatibility and barrier-crossing capabilities, their practical application may be limited by issues such as significant batch-to-batch variability and insufficient targeting. Existing functionalization methods include bioengineering modification, covalent modification, noncovalent modification, and membrane fusion strategies. By introducing targeting ligands or responsive units, functionalized EVs can significantly improve their tumor delivery efficiency, further expanding their therapeutic applicability.

Following the construction of functionalized EVs, achieving precision delivery at the tumor site also warrants significant attention. According to different delivery endpoints, they can be classified into tumor microenvironment delivery, intracellular delivery into tumor cells, and tumor microenvironment–tumor cell intracellular dual delivery. From the perspective of delivery endpoints, tumor microenvironment delivery mostly relies on single target-responsive strategies. Although it has some efficacy, its therapeutic outcomes are susceptible to target expression heterogeneity and nonspecific activation. In contrast, most intracellular delivery into tumor cells and tumor microenvironment–tumor cell intracellular dual delivery relied on dual- or triple-delivery strategies. By integrating multiple levels of different targets and designing logically controllable sequential responses, these strategies are expected to enable more precise tumor therapy.

Despite these advances, several challenges must be addressed before achieving clinical application in tumor therapy. From the perspective of the functionalized EV construction process, the main concerns involve the consistency of EV sourcing and the stability of modifications. Simultaneously, clarifying the endogenous active ingredients of EVs themselves serves as the pharmacological basis for actual medical application. The functionalization process requires establishing unified quality and evaluation standards, as well as detailed in vivo tissue distribution, pharmacokinetics, and safety studies, to provide a reliable basis for clinical use. To ensure safety and therapeutic efficiency, multi-target sequential responsive delivery systems should be optimized, and scalable GMP-compliant production processes should be developed to promote the clinical application of functionalized EVs in precision tumor therapy.

In conclusion, functionalized EVs are a frontier technology in the field of tumor-targeted therapy. Interdisciplinary research integrating biology, chemistry, and engineering can help overcome current problems and is important for promoting the clinical translation of functionalized EVs. Furthermore, functionalized EVs serve not only as excellent drug carriers but can also act as membrane modification modules combined with other materials to further broaden their scope of application, presenting excellent application prospects in biomedicine.

## Figures and Tables

**Figure 1:**
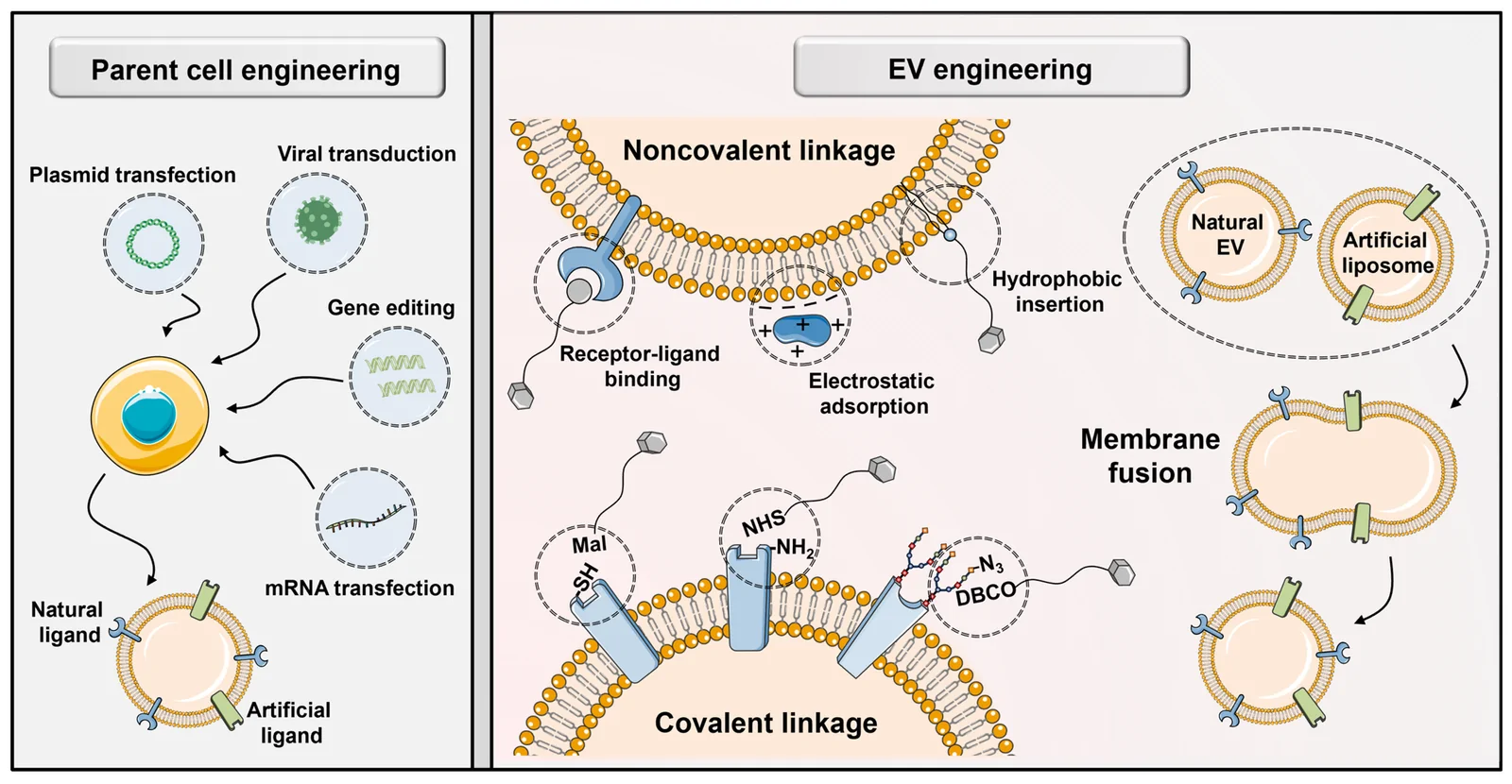
Schematic diagram of the functional modification of EVs.

**Figure 2:**
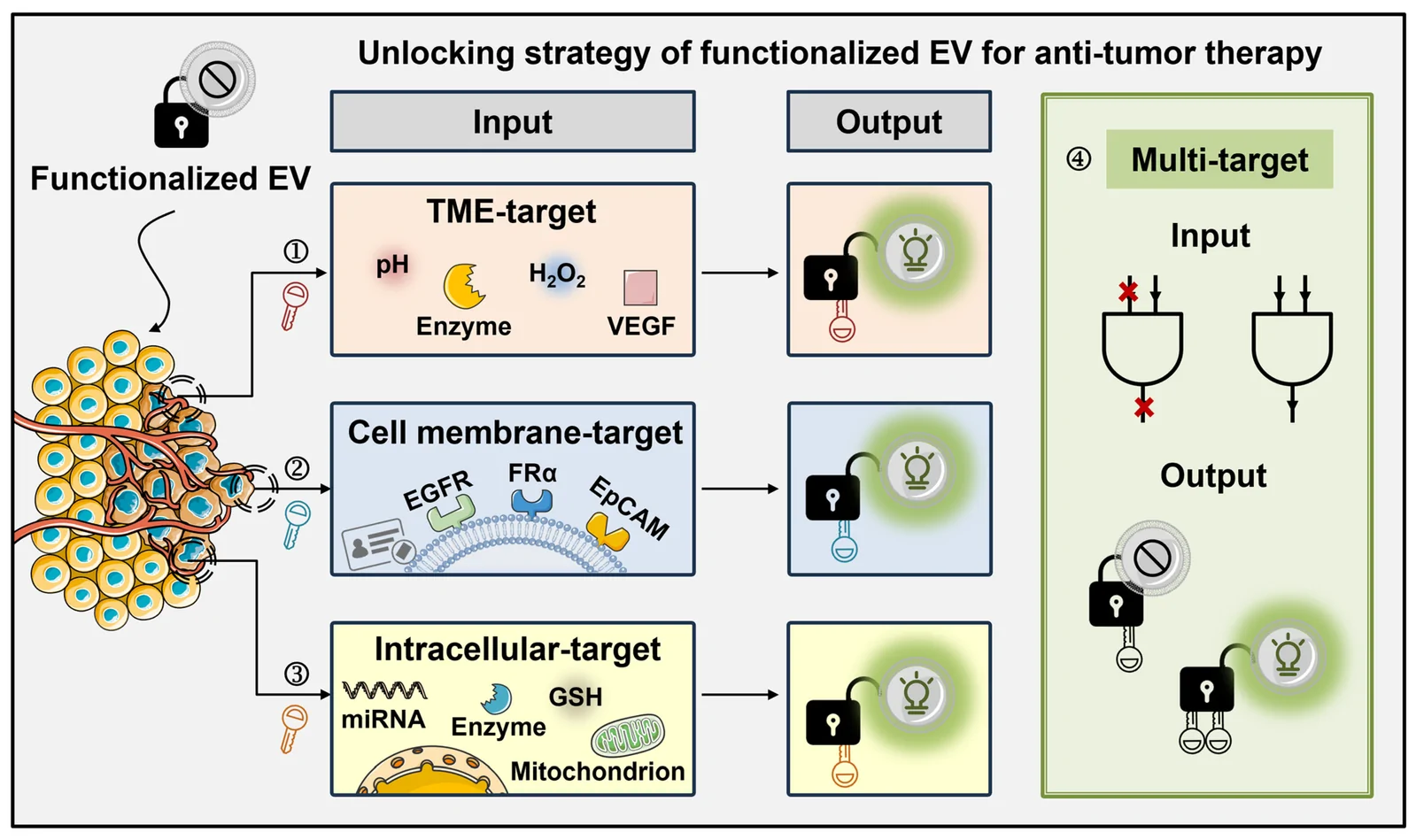
Schematic diagram of the targeted recognition strategies of functionalized EV for antitumor therapy.

**Table 1:** Representative bioactive components and applications of EVs from different sources.

Source	Bioactive Component	Application	Ref.
Turmeric	Curcumin	Full-cycle lung cancer immunotherapy; Wnt/β-catenin inhibition; enhancing immune infiltration by 6.1-fold	[39]
T lymphocyte	miR-765	Inhibit endometrial cancer progression; modulate EMT	[40]
B lymphocyte	CD39, CD73	Suppress CD8^+^ T cell responses and impair the antitumor efficacy of chemotherapy	[41]
Natural killer cell	Granzyme A, granzyme B, perforin, IL-15	Treatment of neuroblastoma, breast cancer, non-small cell lung cancer	[42,43,44,45]
Tea flower	Polyphenol, flavonoid	Inhibition of metastatic breast cancer and lung metastasis; induction of elevated intracellular ROS, superoxide anion, and NO in tumor cells	[46]
Dendritic cell	MHC molecules, CD9, CD11c	Activation of antitumor T cells/NK cells; anti-infective therapy	[47]

**Table 2:** Construction of functionalized EVs and representative applications in disease therapy.

Modification Method	Source	Decorative Component & Loaded Drug	Disease	Stability	Encapsulation Efficiency	Drug-Loading Capacity	Ref.
Bioengineering modification	HEK-293T, GD2-293T	Anti-GD2 nanobody-GPI-modified & DOX-loaded	Osteosarcoma	/	/	DOX: 34.19% ± 1.05%	[60]
Bioengineering modification	HEK-293T	Ephrin-B2-LAMP2b-modified & drug-free	Ovarian cancer	/	/	/	[61]
Covalent modification	Grapefruit	Aptamer LA1 & Psi-LA1-conjugated	Colon cancer	Maintained a stable particle size in 10% FBS for 96 h	/	/	[62]
Covalent modification	Lemon	Heparin-cRGD- modified & DOX-loaded	Ovarian cancer	/	/	/	[63]
Covalent modification	Milk	Anti-EGFR/PD-L1 nanoantibody modified & loaded with miR-21 inhibitor	Triple-negative breast cancer	Zeta potential: toward more negative values	/	36%	[64]
Covalent modification	MCF-7, MDA-MB-231	TPP modified & loaded with DOX/CAR	Breast cancer	Maintained structural stability at 4 °C without aggregation	/	DOX: 9.2 ± 1.1% Carvedilol: 7.6 ± 0.8%	[65]
Noncovalent modification	Hepa 1-6	HA-modified & DOX-loaded	Hepatocellular carcinoma	DOX leakage: 51% (pH 7.4, 24 h)	/	/	[66]
Noncovalent modification	M1 macrophages	RS17 peptide modified & loaded with SR780Fe/REV	Breast cancer	Slight fluctuations in particle size, zeta potential, and PDI after 7 days in FBS	/	/	[67]
Noncovalent modification	HEK293T	IRGD modified & loaded miR-150-3p	Melanoma	Good dispersion; stable particle size	86.4 ± 3.7%	/	[68]
Membrane fusion	Ginseng	Ginseng-modified autologous tumor hybrid membrane	Breast cancer	Minimal particle size variation and no aggregation in PBS within 3 days	/	/	[69]
Membrane fusion	Brain microvascular endothelial	Cubic liposomes fuse & loaded with macromolecules	Brain tumors and neurodegenerative diseases	Stable after storage at room temperature for 3 weeks	/	DOX > 85%, IgG > 85%	[70]

Note: “/” indicates that no result was found.

**Table 3:** Representative functionalized EVs and their therapeutic efficacy based on tumor cell membrane or intracellular single-recognition delivery strategies.

Single- Recognition Delivery Strategy	EV Source	Modifying Component	Model	Therapeutic Efficacy	Ref.
Tumor Cell Membrane	Bovine milk	PE-FA-inserted mExo	Colon cancer	Tumor inhibition rate of ~64.5%; significant increase in TUNEL-positive staining; significant reduction in tumor weight	[55]
Tumor Cell Membrane	BMMSCs	Anti-HER2 VHH conjugation	Breast cancer	Significant decrease in SUVmax and TMR; tumor volume reduction	[83]
Tumor Cell Intracellular	Milk	Cisplatin loading	Ovarian cancer	Compared with the control group, tumor volume decreased from 700 mm^3^ to 300 mm^3^	[84]
Tumor Cell Intracellular	HEK293T	Survivin siRNA-loaded	Oral squamous cell carcinoma	Knockdown of survivin mRNA expression by approximately 60%	[85]

**Table 4:** Representative functionalized EVs and their therapeutic efficacy based on tumor microenvironment-mediated dual-recognition precision delivery strategies.

Dual-Targeted Recognition Strategy	EV Source	Modifying Component	Model	Therapeutic Efficacy	Ref.
TME & cell membrane	Milk	KN035 nanobody & 7D12 nanobody	Colon cancer	Enhanced antitumor efficacy with a tumor inhibition rate of 85.7%; reprogramming of TAMs and enhancement of T cell immunity, with the TAM phagocytic index increasing from 11.0 to 26.7	[64]
TME & cell membrane	M1-type macrophage	pH-sensitive linker & anti-CD47 antibody	Breast cancer	Antitumor; tumor M1/M2 ratio: ~0.1 to ~14; enhanced phagocytosis: 73% of M1 Exo-treated M2 engulfed 4T1 cells	[95]
TME & lysosome	Glioma cell	EVs homotypic targeting & lysosomal acidic environment-responsive	Glioma	Inhibit glioma growth	[96]
TME & endoplasmic reticulum	Bovine milk	KDEL peptide & celastrol (CEL) & PD L1 siRNA	Colorectal cancer	Increase CD8+ T activation and infiltration, CD8^+^ T cells in tumors reached 44.4%, and CD8^+^ T cells in the spleen reached 31.1%; enhance antitumor immunity, 80.0% survival rate	[97]

**Table 7:** A comparative analysis based on different precise delivery endpoints of EVs: design strategies, clinical translation potential, advantages, and limitations.

Delivery Endpoints	Design Strategies	Clinical Translation Potential	Advantages	Limitations	Ref.
TME	Targeting TME components, modulating the immunosuppressive microenvironment	PhaseI/II NCT05375604, NCT04592484	Improved drug accumulation and bioavailability; non-cellular uptake; strong bystander effect	Drug diffusion and loss; intratumoral heterogeneity	[64,92,94,109]
Intracellular	Exploiting organelle-specific signals or physicochemical properties for precise subcellular localization	Predominantly preclinical, Early Phase I	High specificity; overcoming drug resistance; reduced systemic toxicity	Multiple cascading barriers; low endosomal/lysosomal escape efficiency	[66,93,110,111]
TME-Intracellular	Dual regulation of TME-triggered release and precise subcellular delivery	Preclinical	Favorable biosafety profile; enhanced antitumor efficacy; reduced off-target effects and premature leakage	Challenges in clinical translation; stringent requirements for responsive triggering conditions	[104,112]

## Data Availability

No new data were created or analyzed in this study. Data sharing is not applicable to this article.
